# Effectiveness of Information Processing Strategy Training on Academic Task Performance in Children with Learning Disabilities: A Pilot Study

**DOI:** 10.1155/2017/6237689

**Published:** 2017-04-23

**Authors:** Sutinun Juntorn, Sarinya Sriphetcharawut, Peeraya Munkhetvit

**Affiliations:** Department of Occupational Therapy, Faculty of Associated Medical Sciences, Chiang Mai University, Chiang Mai, Thailand

## Abstract

Learning disabilities (LD) can be associated with problems in the four stages of information processing used in learning: input, throughput, output, and feedback. These problems affect the child's ability to learn and perform activities in daily life, especially during academic activities. This study is a pilot study aimed at investigating the effectiveness of information processing strategy training using a combination of two approaches that address the ability to apply processing strategies during academic activities in children with LD. The two approaches are the Perceive, Recall, Plan, and Perform (PRPP) System of Intervention, which is a strategy training intervention, and the Four-Quadrant Model (4QM) of Facilitated Learning approach, which is a systematic facilitator technique. Twenty children with LD were assigned to two groups: the experimental group (*n* = 10) and the control group (*n* = 10). Children in the experimental group received the intervention twice a week for 6 consecutive weeks. Each treatment session took approximately 50 minutes. Children in the control group received traditional intervention twice a week for 6 consecutive weeks. The results indicated that the combination of the PRPP System of Intervention and the 4QM may improve the participants' ability to apply information processing strategies during academic activities.

## 1. Introduction

Learning disability (LD) is a neurological disorder that affects the brain's ability to process, store, and respond to information [1]. According to the Diagnostic and Statistical Manual of Mental Disorders, Fourth Edition, Revised (DSM IV-TR) [2] the term “LD” is a label given to a person who obtains considerably low scores on individually applied standardized tests appropriate for his/her chronological age, grade, and intellectual level. The tests cover reading, mathematics, and written expression skills [2]. Presently, LD is seen as one of the most significant limitations that obstructs participation in school and particularly in learning activities. In the USA, children with LD are the largest category of students receiving special education services. In Thailand, the latest study on children with LD reported that five percent of Thai children aged between six and 12 years (approximately 777, 250 children) have learning disabilities [3].

Occupational therapists use various models to inform intervention with the aim of improving the performance of children with LD. The information processing theoretical model is one model of cognition used by occupational therapists to guide education programing for the children in this group. Information processing is concerned with the stages of processing that supports learning throughout child development and contributes to purposeful and effective sensing and thinking skills, making performance quick and easy [1]. The first stage of processing comprises registration and attention to sensory information which is important to task performance [4, 5]. The second stage in processing is memory. Working memory (short-term memory) and long-term memory strategies are used to assign meaning to sensory information registered from the environment together with a store of knowledge from past experiences to enable children to predict what will happen next and direct the appropriate action. Children with LD are thought to have delayed processing capacity leading to reduced memory, characterized by inefficient storage and retrieval strategies [6]. They have a reduced ability to quickly recognize the sensory data such as objects, letters, words, and procedures previously learned and therefore require more repetition than their typical peers. The following stage of the information processing includes planning, organizing, and problem solving. This process is required for organizing multiple tasks and parts of tasks into a seamless whole [4]. More importantly, it also allows them to respond to new information or engage in new and complex learning to solve problems and to evaluate thinking, leading to independent learning. Children with LD often have problems with planning and problem solving. They often have difficulty in preparation and planning, even if they have good memory [7]. Research demonstrates that children with LD are unable to self-instruct as well as their typical peers [1]. The last stage of information processing is processing output and feedback. Independent learning is dependent not only upon efficient processing throughout the system, but also on the capacity to process the feedback information regarding their performance. Children who have reduced planning have trouble judging and monitoring responses resulting in difficulties in independent learning [1]. Previously, research has been conducted into effective ways to assist children with LD to perform in school learning. The methods used to help children process information for learning involves teaching them to use specific thinking strategies while engaged in sophisticated novel tasks. Many problem solving approaches have been used to develop the ability of information processing while children perform tasks. The unique characteristic of this approach shifts from direct instruction to the instruction of processing strategies during task performance [8].

Information processing employs a number of different processing strategies that are organized, integrated, and adapted to allow occupational performance. Efficiency depends on the ability to select the most appropriate processing strategies that result in “efficient use” of information processing [9]. Cognitive strategies are ways of thinking or the ability to organize thoughts in a goal-directed way in order to accomplish a goal [10]. Cognitive strategy use implies salience or use of known cognitive strategies in the “here and now.” It is the ability to choose and apply the “best” strategy to fit a particular situation. Effective cognitive strategy use has been defined as the simplest and most efficient means of processing information relative to a situation and enables a person to participate in every activities without having to “think too hard” [11]. Reduced cognitive strategy use is different to implying the presence of reduced cognition per se in children with LD. While the majority of children with LD have typical cognitive capacity, their ability to “use” their cognition for function is thought to be compromised by their learning disorder [12]. Cognitive strategy training approaches are one of the ways that have been used to promote the capabilities associated with the use of processing strategies or cognitive strategies to improve academic performance [13]. This approach focuses on the acquisition and application of strategies to process information more efficiently. While performing the task, the children need cognitive strategies to perceive the nature of a task in the context of the environment, the recognition of similarities between the present tasks and past experiences. They must also be aware of task relationships and jobs, must direct goal expansion, engage in goal formation, and make decisions on their performance. The skill acquisition that resulted from the intervention in the form of strategy training approach helps children's ability to generalize functional performance in other environments [14]. Besides cognitive strategy training, systematic instruction approach can also enhance occupational performance by focusing on developing specific skills required for occupational performance [15]. Systematic instruction is one of the techniques of the functional task or behavioral approaches; it is a top-down approach similar to strategy training. The intervention used in this approach focuses on task analysis, cueing, prompting and chaining, support, and guiding task performance [16, 17]. These are consistent with an acquisition frame of reference based on learning theory [18]. The primary goal of this manner of intervention is to focus on the learner's ability to function according to the steps of the specific task to achieve skill acquisition in selected tasks during the treatment [16].

Most recently, Chapparo and Ranka [8] have developed an intervention approach that integrates strategy training, systematic instruction, learning theory, and information processing theory calling it the Perceive, Recall, Plan, and Perform System of Intervention (or PRPP System of Intervention). The PRPP System of Intervention was developed from the PRPP System of Task Analysis, which is a dynamic assessment. The uniqueness of this intervention is that it is an occupation-based practice. The PRPP System of Intervention aims to improve task performance mastery by addressing the specific cognitive strategy application behaviors identified through the PRPP System assessment that most impacts successful mastery. Strategy instructional methods used to address these behaviors incorporate cues and prompts that align with specific Perceive, Recall, Plan, and Perform processing operations and preferences. During the PRPP System of Intervention, the prompts of “Stop, Attend, Sense, Think, and Do” are initially used as content-free “metaprompts” to alert children to process information required for task performance. These global prompts are followed up with more specific content based behavioral prompts based on findings from the assessment component of the system [4]. The “Attend/Sense” strategy instruction addresses the perception problem that comes from poor attention to sensory images and lack of focus on the tasks. The children are stimulated to receive and encode information of activities until completed. The “Think” strategy instruction can help children enhance recall skills when they could not recognize sensory images or made mistakes with recognition, naming things incorrectly, or forgetting about the object's instruction. This strategy instruction focuses on repetition and organization which are important strategies for keeping information in working memory [4]. Moreover, the “Think” strategy instruction also addresses the planning strategy problem by letting intervention focus on the child's ability to construct and evaluate their own goal-oriented strategies for action before performing tasks, especially the more novel or complex ones. When children use thinking strategy effectively, they can generalize performance to another situation. The “Do” strategy instruction helps children to start and stop. Moreover, this strategy is able to stimulate continuing, persisting, and flowing with the task until it is finished [4]. The efficacy of this approach has been observed in children and adults with a variety of diagnoses and conditions that impact everyday cognition [4] and also has been demonstrated through research with adults with traumatic brain impairment [19] and children with social and behavioral difficulties [20]. However, in order to effectively use the strategy training in the PRPP System of Intervention, the therapist needs to respond to the needs of each student and adjust the strategies to be in line with those needs. Moreover, one application of evidence based principles for systematic instruction of the PRPP System of Intervention involves ensuring that therapists consider the prompts, which are more behavioral and specific content based, by using the least to most prompt hierarchy [4]. Therefore, it is necessary for the therapists to have a guideline of systematic facilitation to serve as a scaffold for organizing the selection and implementation of effective teaching and learning strategies relevant to the needs of each child. In the past a study was conducted by Vygotsky [21], which found that children could develop advanced abilities through guidance from a facilitator to achieve autonomy through the use of systematic facilitation called “scaffolds.” This concept involves the therapists being the motivators to the children who then motivate themselves. Additionally, the use of a prompts starts in the overt to covert manner. That aim is consistent with a study conducted by Mosston and Ashworth [22] that proposed that the nature of teaching will start from command style to self-teaching. The command style is a direct style that aims to teach learners about the key elements or how to respond to the demands of the task. The self-teaching style encourages learners to consider the barriers to performing the activity and how to best deal with them. This allows learners to use better decision-making processes.

**Figure 1:**
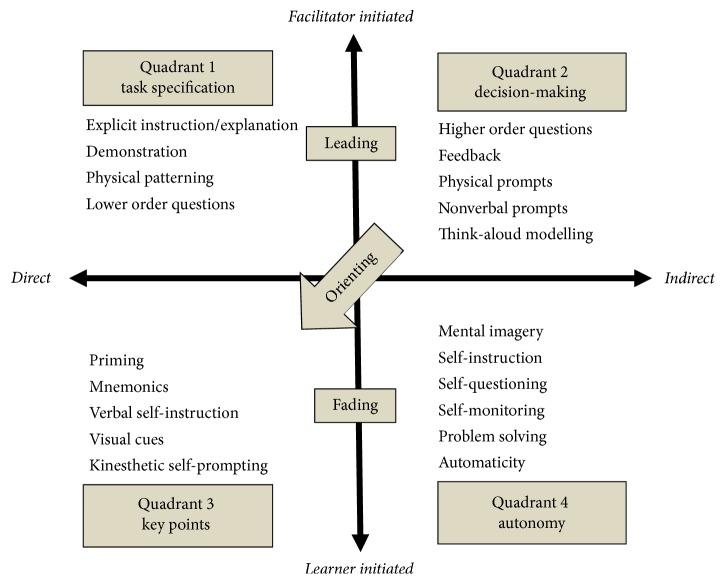
The Four-Quadrant Model of Facilitated Learning [modified from [18]].

The Four-Quadrant Model (4QM) of Facilitated Learning [18] is another approach that allows therapists to understand, plan, and coordinate the use of specific teaching and learning strategies by means of systematic facilitation. The 4QM was considered to be a suitable framework that could be used together with the PRPP System of Intervention in this study. The 4QM groups the various cognitive and physical learning strategies that children can apply to perform occupation tasks more autonomously. The use of prompts in the 4QM moves from the facilitator to the child. Also, the strategies that are more direct and allow learning about the characteristics of the task or the responses required combined with the indirect strategies enable learners to improve decision-making processes for better results. In this group the students are active in planning, executing, and evaluating performance. The 4QM would be relevant to occupational therapists that regularly facilitate skill acquisition as part of their service offering. It has been suggested as one way of informing the selection of effective learning strategies based on the changing needs of the learner when acquiring a new skill. Two continua integrations are used with the 4QM: (i) strategy directness and (ii) clustering the initiation source to prompting to group learning strategies according to the learner's needs. Numbers are used to identify the clusters. Quadrant 1 directly facilitates the prompting initiation that specifics the task and/or response, and indirect facilitator-initiated prompts encourage decision-making. In Quadrant 2, the learners recall that the use of overt self-prompt is encouraged. In Quadrant 3 autonomous performance is supported by the learners through self-regulatory cognitive and metacognitive strategies grouped in Quadrant 4 [23]. The Four-Quadrant Model of Facilitated Learning is shown in Figure 1.

The 4QM strategies had been used in a case study on four children and the PRPP System of Task Analysis was used to a measure the outcomes. The results showed that after the intervention the PRPP scores increased by 58–76% [24]. Moreover, the study of perspectives of the occupational therapists who had experience in working for seven or more years on the clinical utility of the 4QM process through focus groups found that participants can be made aware of the advantages of the 4QM. This helps guide therapist to respond to the needs of the learner with regard to their personal abilities and learning styles [25].

To apply the 4QM coupled with the PRPP System of Intervention, the PRPP System of Task Analysis was used by therapists in finding problematic descriptors across all PRPP System Quadrants. Next, the problematic descriptors were chunked to set a goal and plan the intervention. Afterwards, the 4QM, which focuses on using a systematic prompting appropriate for each child, was used in combination with the PRPP System of Intervention. This focuses on children learning to use a sequence of processing strategies to “Stop/Attend, Sense, Think, Do” so that children can use more efficient strategies to improve performance and autonomy.

However, the PRPP System of Intervention and the 4QM have never been used in children with LD. Thus, the aim of this study was to investigate the effects of information processing strategy training using a combination of two approaches for children with LD.

## 2. Methodology

### 2.1. Participants

Twenty children with LD were recruited from schools in Chiang Mai province, Thailand, by purposive sampling method, and were allocated to the experimental group (*n* = 10) or the control group (*n* = 10). All of them had reading disabilities and had received traditional intervention such as visual perception activities, visual-motor activities, sensory-motor activities, cognitive activities, and handwriting activities twice a week. The age range of the participants in the study was from 10 to 12 years. Participants were attending fourth to sixth grade in six inclusion schools in the broader area of Chiang Mai province, Thailand, which had children diagnosed with learning disabilities. The inclusion criteria were as follows: firstly, children could communicate without visual, hearing, and speaking impairments that could be traced back to the children's personal clinical profile. Secondly, parents had to agree to sign an informed consent to participate in the study. The participants who could not complete the intervention program were excluded.

### 2.2. Outcome Measures

**Figure 2:**
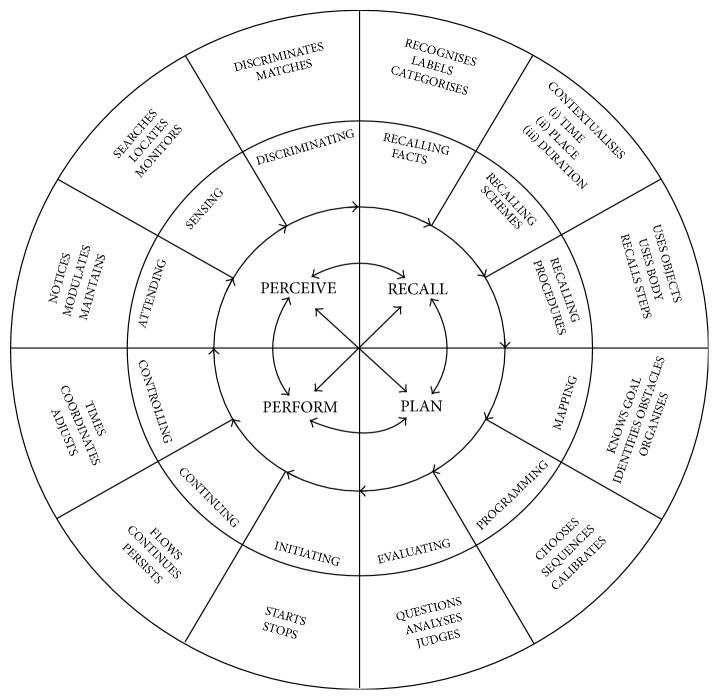
The PRPP System of Task Analysis: conceptual model of information processing behaviors [cited in [14]].

The outcome measure used in this study was the Perceive, Recall, Plan, and Perform System of Task Analysis: Thai Version or PRPP System: Thai Version. The PRPP System: Thai Version is the Thai version of the original Perceive, Recall, Plan, and Perform (PRPP) System of Task Analysis which was developed by Chapparo in 2010 [4]. It is one of the occupation-focused assessments that measures cognitive strategy use in a specific context [8]. It is composed of two assessment stages. Stage One Analysis employed a standard behavioral task analysis to indicate the person's mastery for specific and relevant occupations. Stage Two Analysis adopts a cognitive task analysis which identifies the effectiveness of cognitive strategy use during task performance. This stage of the PRPP System of Task Analysis is conceptually divided into four quadrants: Perceive, Recall, Plan, and Perform. Each quadrant is broken down into three subquadrants and several underlying information processing strategies termed “Descriptors” as shown in Figure 2. Descriptor behaviors are rated by an observer on a 3-2-1 scale as follows [8]: 3 scores = a participant will receive 3 scores when the task is completed safely without assistance and prompts and using reasonable time; 2 scores = a participant will receive 2 scores when the task is completed safely, but some concern and deficit in this behavior are noted. Prompts may be needed and time is not restricted to receive this score. Last, a participant will receive a score of 1 when the task is uncompleted and student has demonstrated a behavioral deficit. Participants may act unsafely during completing the tasks, taking too much time or requiring too many prompts to receive this score.

The PRPP System of Task Analysis has standardized administrative procedures relative to language of the assessment, method of observation, and scoring. It has reported content, discriminant, cultural and concurrent validity, internal consistency, interrater reliability (occupational therapist-occupational therapist), and test-retest reliability [26–32]. For use in the PRPP System, the Thai Version was examined for its reliability and validity in Thai patients with acquired brain injury and showed high test-retest and interrater reliability [33]. The PRPP System: Thai Version was preliminarily used with Thai patients with stroke [34] and persons with schizophrenia [35].

### 2.3. Intervention Program

The intervention program in this study was based on the integration of the concept of the PRPP System of Intervention and the 4QM of Facilitated Learning program. As the PRPP System of Intervention broadens the traditional focus of programmed learning and behavioral task analysis by augmenting information processing strategies upon the cognitive process being employed while learning, the intervention sessions specifically targeted application of information processing strategies using graded prompts, progressing from content-free metaprompts to more specific content based behavioral prompts. The participants learn to apply* Stopping, Sensing, Thinking, *and* Doing* strategies to their performance during performing tasks. The PRPP Core Intervention Strategies are explained in Table 1.

The PRPP System of Intervention integrates aspects of a systematic instruction and information processing theory. It is a task-oriented information processing approach that simultaneously focuses on task training, strategy training, and strategy application within the context of everyday performance [8]. To enable children to apply a sequence of processing strategies orders, which are “Stop, Sense, Think, and Do,” they have to use prompts in the correct sequence. The suitable prompts can stimulate information processing required for task performance. The 4QM of Facilitated Learning is a program to provide a framework for therapists to understand teaching and learning processes. To use this model the therapists choose a strategy for using prompts, which matches the learners' requirements. It enables them to show their skills and improve performance in an autonomous way [23]. Each of the strategies in the 4QM involves methods that enable learning by using the backward method to find the beginning of the intervention. Although each of the participants in this study started by using different ways to initiate the process of the activity, ultimately after finishing the intervention program, most of the participants could use the strategies autonomously. Therefore to integrate these two approaches, the therapist needs to detect the impairment of the information processing strategy application during the activity by using the PRPP System of Task Analysis. After the therapist detects the dominant deficiency in the descriptors of each quadrant of the PRPP System in each child, the problematic descriptors would be set as a goal for intervention. Next, the 4QM strategies, which focus on using a systematic prompt appropriate on each child, were used together with the PRPP System of Intervention. This focuses on children learning to use a sequence of processing strategies to “Stop, Attend, Sense, Think, and Do” so that children can use more efficient strategies to autonomously perform tasks.

To understand how the combination of the PRPP System of Intervention and the 4QM approach was implemented in this study, an example of how to use this program is shown in Table 2.

The following are examples of how the 4QM and the PRPP System of Intervention work together in one case study. After evaluating the abilities of the child using the PRPP System of Task Analysis, it was found that the child had problems on descriptors such as Modulates, Maintains, Searches, Locates, Recalls Steps, Analyses, Judges, Flows, and Continues. The intervention for improving “Modulates” strategy involves the therapist holding the child's finger to help the child move to the text word by word. This is a physical patterning strategy in Quadrant 1 of the 4QM. At the same time, the therapist used a verbal prompt “Stop, Look at your page, Focus on the first word…Shift your eyes to the second word!” This is a strategy used in the process “Stop/Attend” in the PRPP System of Intervention. Such techniques can help children when reading and learning new vocabulary change their focus to the next word. Later, when the children can slide a finger to terminology correctly, the therapist then reduces the aid by using a feedback strategy in Quadrant 2 of the 4QM with the comment “You slide too early!” This technique allowed the child to use self-regulatory procedures. Next, the child applied a visual cue strategy in Quadrant 3 of the 4QM by using a red pen to divide the word in order to see it more clearly. Finally, the child uses a self-monitoring strategy in Quadrant 4 of the 4QM to determine whether or not they read the words.

The intervention used in the “Maintains” strategy involves the therapist using verbal prompts such as “Keep concentrating until you have finished the sentence!” which is a strategy used in the process of “Stop/Attend” of the PRPP System of Intervention. At the same time, the therapist tapped on the line where the child read, which is a nonverbal prompt strategy found in Quadrant 2 of the 4QM, designed to keep kids focused on the task. Later, when the child is more focused, the therapist guides the child to use a kinesthetic self-prompting found in Quadrant 3 of the 4QM by tapping along the line while browsing to redirect eye gaze. Doing so helps enhances direct attention of the reader. Ultimately, the child could use a self-monitoring strategy found in Quadrant 4 of the 4QM to enhance constant attention to task.

During the “Searches” and “Locates” strategies, the therapist uses verbal prompt such as “Get your eyes ready to look for your page!” which is a strategy used in the process of “Sense” of the PRPP System of Intervention. Afterward, the therapist guides the child to highlight vocabulary which is a keyword in the question to find the answer in the article. This strategy is a visual cue mentioned in Quadrant 3 of the 4QM. Lastly, the child could use a self-instruction strategy found in Quadrant 4 of the 4QM to find the answer.

The intervention of “Recalls Steps” strategy starts with a verbal prompt “Remember what you did yesterday!” This is a strategy used in the process of “Think” of the PRPP System of Intervention. Next, the therapist guides the child to use a mnemonics strategy in Quadrant 3 of 4QM where the child reminds herself to recall the steps involved in performance by saying the keywords in the task steps. It starts with reading the passage and choosing a word to fill in the blanks. Afterward, the question is read and the answer is written. Ultimately, the child engages in covert self-instruction in Quadrant 4 of the 4QM until the task was completed.

The intervention of “Analyses” and “Judges” strategies starts from an explicit instruction and explanation in Quadrant 1 of the 4QM by writing the words on the board, along with reading and explaining the meaning of the word. This strategy can then involve filling in gaps to make a complete sentence. This method can teach the child to learn the right information. Next, the therapist uses a verbal prompt such as “Ask yourself, does this look right?” and “Which word should be filled in the blank?” This strategy is used in the process of “Think” of the PRPP System of Intervention. After that, the therapist guides the child to use a verbal self-instruction strategy found in Quadrant 3 of the 4QM to make decisions necessary to write the right word. The child says to herself “Is this word correct?” “Can another word be used instead?” Lastly, the child uses self-questioning and self-monitoring strategies found in Quadrant 4 of the 4QM to critique their plan of action.

The intervention of “Continues” and “Flows” strategies starts with a verbal prompt “Keep going until you reach the last sentence.” This is a strategy used in the process of “Do” in the PRPP System of Intervention with a finger pointing to the line that the child is reading, a strategy of nonverbal prompt found in Quadrant 2 of the 4QM. After the child could operate continuously, she reminded herself by marking at the end of the line where she had completed the self-instruction to start a new line. This method is a visual cue strategy in Quadrant 3 of the 4QM. At the end, the child could use a self-monitoring strategy in Quadrant 4 of the 4QM until the task is completed.

### 2.4. Procedure

The proposal was sent to the Ethics Committee of the Faculty of Associated Medical Sciences, Chiang Mai University, Thailand, for approval. Then researcher asked for permission from the directors of each school and contacted teachers who worked with special needs children. Twenty children with learning disabilities were then recruited according to the predetermined criteria. After informed consent was obtained from participants' parents, all participants in the experimental group and the control group were asked to perform academic activities (reading comprehension and written expression). Each child was asked to read the passages which were designed for students at the fourth- to sixth-grade reading level. Next, the child filled in the appropriate words in the blanks to complete the sentences and article. Finally, the child was asked to answer the questions that followed. The criteria for specifying these activities were based on the PRPP System of Task Analysis: Thai Version [9]. The reading comprehension and written expression tasks were selected in the assessment process as they were considered important and meaningful for the participants' roles as students. The PRPP System of Assessment: Thai Version was done by a trained occupational therapist who was a blind assessor. Scores obtained from this stage were pretest scores.

All participants were then randomly assigned to the experimental (intervention) group (*n* = 10) or the control group (*n* = 10). The experimental group received the intervention program in addition to the traditional program twice a week for 6 consecutive weeks. Each session in the intervention program took approximately 50 minutes. The control group received traditional intervention twice a week from occupational therapists in the Rajanagarindra Institute of Child Development, Chiang Mai province, Thailand, only. The traditional programs of the control group kept the focus on the deficits of components of function. These programs were not aspects of occupation-based practices. The traditional programs focused on the activities of visual perception activities, visual-motor activities, sensory-motor activities, cognitive activities, and handwriting activities. Examples of activities used in the practice of the control group based on traditional programs are shown in Table 3.

After the intervention period was over, all participants in both groups were reassessed using the PRPP System: Thai Version by the same assessor. Scores obtained from this stage were posttest scores.

### 2.5. Data Analysis

1. The pretest and posttest scores obtained from Stage Two Analysis of the PRPP System: Thai Version were computed to compare scores between the intervention and the control group using the Mann–Whitney *U* Test. The significant level was set at .05.
2. The pretest and posttest scores obtained from Stage Two Analysis of the PRPP System: Thai Version were computed to compare scores within the group using the Wilcoxon signed-ranks test. The significant level was set at .05.
3. Effect sizes (ES) for mean differences were calculated by calculating for nonparametric tests [36]:
    Mann –Whitney:
   
*r* = *Z*/square root of *N*, where *N* is total number of cases that will have *Z* values reported.
    Wilcoxon signed-ranks test:
   
*r* = *Z*/square root of *N*, where *N* is number of observations of two times points.

## 3. Results

### 3.1. Demographic Data

The demographic characters of the samples are presented in Table 4. In the control group, the average age was 11.33 years. For the experimental group, the average age of the participants was 11.06 years. The majority of the participants in both groups were male.

### 3.2. The Comparison of Quadrant and Subquadrant Pretest Scores between the Control and Experimental Group

From Table 5, the Mann–Whitney *U* test analysis results indicated that the pretest scores of all quadrants and subquadrants showed no significant difference between the control and the experimental group except in the Mapping Subquadrant of the Plan Quadrant (*p* > .05).

### 3.3. The Comparison of Quadrant and Subquadrant Pretest and Posttest Scores between the Control and Experimental Group

From Table 6, for the control group, the pretest and posttest scores in all quadrants and subquadrants (except the Sensing Subquadrant in the Perceive Quadrant) demonstrated no significant difference (*p* > .05). For the experimental group, the pretest and posttest scores in all quadrants and subquadrants (except the Attending and Discriminating Subquadrants in the Perceive Quadrant, Recalling Schemes Subquadrant in the Recall Quadrant, and Initiating and Controlling Subquadrants in the Perform Quadrant) demonstrated significant difference (*p* < .05).

### 3.4. The Comparison of Quadrant and Subquadrant Posttest Scores between the Control and Experimental Group

From Table 7, the analysis results from the Mann–Whitney *U* test indicated that the scores of the Perceive, Plan, and Perform Quadrants showed a significant difference between the control and the experimental group at posttest (*p* < .05, *p* < .001). The Recall Quadrant showed no significant difference (*p* > .05). However, there were two subquadrants in the Recall Quadrant (Recalling Facts and Recalling Procedures) that demonstrated a significant difference between the two groups (*p* < .05).

## 4. Discussion

The purpose of the present study was to investigate the effectiveness of the combination of the PRPP System of Intervention and the 4QM approach on the academic activities (reading comprehension and written expression) of children with LD. The overall finding of the study demonstrated that after the intervention program the experimental group significantly improved in the mean scores of all PRPP System Quadrants (Perceive, Recall, Plan, and Perform), whereas the control group did not show a significant improvement in all quadrants. There may be several reasons for this finding. Firstly, the intervention program focused on facilitating multiprocess sequences of strategies. Since the nature of information processing is dynamic and employs a number of different cognitive strategies [9], the cognitive strategy training that is effective must stimulate multiple cognitive processes rather than individual cognitive skills without focusing on the interrelationships between specific cognitive skills for performing tasks [37]. The intervention program in this study focuses on the optimization of the problematic strategies that have been “chunked” together in each quadrant of the PRPP System of Task Analysis. The therapists teach children to learn and apply a sequence of processing strategies to “Stop/Attend, Sense, Think, and Do” with the systematic prompt of the 4QM by the process of attention and sensory perception (Perceive Quadrant), memory (Recall Quadrant), response planning and evaluation (Plan Quadrant), and performance monitoring (Perform Quadrant) to have the flow of information processing from initial input to response output. The effective intervention program of each stage of information processing may occur through the system in the following manner.

In order for the stimulating attention and sensory perception (Perceive Quadrant) to be more efficient, the prompt of “Stop/Attend/Sense” and systematic prompt of the 4QM were used to encourage children to modulate arousal and reallocate attention. This includes the modulation of attention (Attending Subquadrant) and discrimination of sensory information (Discriminating Subquadrant). Improvement of each problematic descriptor score in Perceive Quadrant was due to facilitation on the function of the problematic descriptors during performance of the task. For example, if the children have a problem on “Modulates” strategy, prompting was used to help children shift their eyes in order to encourage them to change the focus of their attention to suit the reading task. The verbal prompt “Focus on the first word!…Shift your eyes to the second word!” which is in the PRPP System of Intervention and the prompting hierarchy of the 4QM were used to enhance the ability to get the appropriate scope for task performance and to allocate the attention to different parts of the tasks. These help children to focus on specific and significant elements of task performance within specific boundaries. The effective “Modulates” strategy improves attention ability (Attending Subquadrant) since it is a part of attention mechanisms, which is the main element of the perceive process. The result is that these intervention programs help children be more effective at gathering sensory behaviors while performing academic activities.

To promote memory (Recall Quadrant), the prompt of “Think to remember” and systematic prompt of the 4QM were used to encourage children to store, retrieve, and use the information necessary to perform tasks effectively. For example, if the children have a problem on the “Recalling Steps” strategy, all of prompts were focused on encouraging the children to remember the sequence of task. The verbal prompt “Remember what you did yesterday,” which is in the PRPP System of Intervention, and the mnemonics strategy of the 4QM were applied to improve the ability to recall information about how to sequence steps needed for task. These techniques are confirmed by Thagard (2005), who described how repetition and organization of instruction can help children to get information into working memory [6]. The effective “Recalling Steps” strategy improved recalling procedures ability (Recalling Procedures Subquadrant), which is the one element of the recall process. Therefore, the intervention program can enhance the recall ability while performing academic tasks.

To promote response planning and evaluation (Plan Quadrant), the prompt of “Think to problem-solve” and the systematic prompt of 4QM were used to encourage children to construct and evaluate their own goal-oriented strategies for action before performing tasks. For example, if the children have difficulties with “Analyses” and “Judges” strategies application, all prompts were employed to help the children to analyze real and potential constraints and act accordingly and to make safe and informed decisions. The verbal prompt “Ask your self does this look right…Which word is more proper to be filled in the blank?” which is in the PRPP System of Intervention and prompting hierarchy of the 4QM should be used to help the children to learn the right information about tasks, evaluate the outcome, and decide how to accomplish the tasks in the most complete manner. The effective “Analyses” and “Judges” strategy improves evaluating ability (Evaluating Subquadrant), reflecting a monitoring and appraisal process. The children can use thinking strategies for planning and problem solving of complex and novel occupational tasks by being able to self-evaluate and decide the need to change or adapt. Finally, children may have a complete idea of the expected outcomes and prepare to put a plan into action.

To promote performance monitoring (Perform Quadrant), the prompt of “Do” and the systematic prompt of the 4QM were used to encourage children to actively respond and process information that requires the ability to plan and initiate the start and stop of action. For example, if the children demonstrate problems with “Flows” and “Continues” strategies, any prompting improves the children's ability to persist in carrying out a plan in a smooth, flowing manner until the step or task is completed. The verbal prompt “Keep going until you reach the last sentence,” which is in the PRPP System of Intervention, and the prompting hierarchy of the 4QM were used to facilitate the children to transfer between tasks and parts of tasks and to complete tasks. In doing so, the continuing process (Continuing Subquadrant) in the performing procedure was developed, as well.

Next, prompting that starts from the therapist to the child is a special feature that is used in the intervention program. This approach was used to minimize reliance on therapists and to promote a quicker transition to independent performance [18]. While implementing intervention, the therapist must find a way to direct a pathway toward autonomy and, at the same time, be flexible concerning the child's response time and the context of the task to be performed. The intervention program that uses scaffolding or a systematic facilitator support is a guideline to help therapists choose the appropriate prompt to enhance occupational performance and to help the student to be able to complete the performance autonomously. Facilitator-initiated methods were used in the early stages for the children to understand the characteristics of the task and the performance required and to help them make decisions about the task. Afterward, the therapist encourages the child to engage and use learner-initiated strategies to select the target recall key points essential to task performance. At this point, the child is able to perform the task autonomously.

Finally, the main characteristic of the intervention program is that it is a top-down approach. Thus, the therapist always responds to any changes in the learner's requirements and initiates an adapting strategy in order to make sure the children can do specific work which is important for them. There are decisions to be made about the development of efficient performance and responses to errors. When the child develops their performance by themselves, they can better transfer knowledge to real-life situations. Furthermore, the child's motivation is the most important factor that affects the child's ability to engage in tasks. Since this combined intervention is client-centered, the expected outcomes are determined by considering the child's occupational roles and context of the learning situation. Children are likely to be more motivated to engage in improving their performance when they identify goals to work on by themselves [38].

In summary, targeting cognitive strategies, facilitating multiprocess sequences of strategies that moved away from therapist to child, and the client-centered nature of the intervention were thought to be the reasons for the success achieved using the combination of the PRPP System of Intervention and the 4QM approach on the academic activities.

*Limitations and Suggestions for Future Studies*. This study showed that applying the combination of the PRPP System of Intervention and the 4QM approach was an effective treatment method to be used with the sample of children with LD. The success of the systematic strategy instruction program was found in the effective information processing strategy applications for treading comprehension and written expression tasks. These findings are worthy of further investigation. However, there are some limitations inherent in this study. First, the sample size was small and the participants were recruited from only Thai schools that supported children who had disabilities. Future research is recommended to apply this intervention program on a larger sample size and from a variety of backgrounds. Lastly, the participants were diagnosed with learning disabilities in reading (dyslexia). Future research should include children with different types of LD. Moreover, studies in the future should apply this program to other sample groups who have problems with information processing application such as ADHD and DCD. Future research that examines the combination of the PRPP System of Intervention and the 4QM approach program should focus on continuing the techniques of this study and a long-term follow-up study should be done on the children who received the intervention program. Since the combination of the PRPP System of Intervention and the 4QM approach is a client-centered approach, analyzing qualitative data of information processing strategy application ability of activities in the samples should be explored before and after receiving intervention from both children and their parents.

## 5. Conclusion

This study evaluated the effectiveness of the combination of the PRPP System of Intervention and the 4QM approach. The combination of these two approaches was shown to be effective in improving learning strategies during the academic activity in Thai children with LD. The experimental group had significantly improved in the mean scores of all quadrants of the PRPP System: Thai Version.

## Figures and Tables

**Table 1:** PRPP Core Intervention Strategies [8].

Strategy	Definition
Intervention goal is task mastery	(i) Expected outcome is improved functional performance in everyday tasks required by the person's occupational roles and context.
	(ii) Intervention success is measured by increased functional performance.

Application of evidence based principle of systematic instruction	(i) Goal of intervention is clear to client.
	(ii) Least to most prompt hierarchy is used.
	(iii) Multiple opportunities for practice of the task and target cognitive strategy are offered and performance errors are prevented.
	(iv) Learning occurs across natural contexts to promote generalization.
	(v) Feedback is specific to task mastery and the cognitive strategy that is the target of intervention.

Cognitive strategies are behaviorally defined and measurable	(i) Strategies required for task performance are identified using the PRPP System of Task Analysis and their effectiveness measured before and throughout intervention.

“Chunking” of strategies across all PRPP Quadrants is planned	(i) Starting with “*Stop,*” one or two strategies are targeted from each processing quadrant for “*Sense*” (Perceive Quadrant), “*Think*” (Recall and Plan Quadrants), and “*Do*” (Perform Quadrant).
	(ii) Single descriptors are not used.
	(iii) A line of processing required for the task mirrors the definition of arrows in the center of the PRPP System of Task Analysis.

Focus of intervention is on application of cognitive strategies (descriptors) to real-world performance	(i) The descriptor behaviors form the verbal, physical, or visual prompts given during performance.
	(ii) The therapist acts as a cognitive mediator.
	(iii) The patient is taught to self-instruct in the strategies.

**Table 2:** The example of using the combination of the PRPP System of Intervention and the 4QM approach for academic activity (reading comprehension and written expression).

Target descriptors/subquadrants/quadrants of PRPP System	Strategies of PRPP System of Intervention	Prompting of PRPP System of Intervention (verbal)	Prompting of 4QM
Modulates/Attending/Perceive	Stop/attend	“Focus on the first word, now shift your eyes to the second word”	(i) Physical patterning, Q1 (ii) Feedback, Q2 (iii) Visual cue, Q3 (iv) Self-monitoring, Q4
Maintains/Attending/Perceive		“Keep concentrating until you have finished the sentence”	(i) Nonverbal prompts, Q2 (iii) Kinesthetic self- prompting, Q3 (iii) Self- monitoring, Q4

Searches/Discriminating/Perceive Locates/Discriminating/Perceive	Sense	“Get your eyes ready to look for your page”	(i) Visual cues, Q3 (ii) Self-instruction, Q4

Recalls Steps/Recalling Procedures/Recall Analyses/Evaluating/Plan Judges/Evaluating/Plan	Think	“Remember what you did yesterday” “Ask your self does this look right? Which the word is more proper to be filled in the blank?”	(i) Mnemonics, Q3 (ii) Self-instruction, Q4 (ii) Explicit instruction/explanation, Q1 (iv) Verbal self-instruction, Q3 (v) Self-questioning & self- monitoring, Q4

Flows/Continuing/Perform Continues/Continuing/Perform	Do	“Keep going until you reach the last sentence”	(i) Nonverbal prompt, Q2 (ii) Visual cue, Q3 (iii) Self-monitoring, Q4

**Table 3:** Example of activities in the traditional program for the control group.

Visual perception activities	Visual-motor integration activities	Sensory-motor activities	Cognitive activities	Handwriting activities
(i) Finding the letters hidden in a ball bowl (ii) Doing pegboard (iii) Connecting the dots to complete the picture (iv) Finding the differences between two pictures	(i) Throwing the ball into the target vowel (ii) Rolling bowls to the target word (iii) Jumping on foam boards as instructed	(i) Moving body parts as the model (ii) A crawl through, climb over obstacles (iii) walking the balance beam (iv) Animal walk	(i) Matching card game (ii) Sequencing card game (iii) Categorize objects	(i) Writing letters on sand (ii) Sculpturing clay in to characters

**Table 4:** Demographic variables of the control and experimental group.

Demographic characteristics	Control group		Experimental group	
	(*n* = 10)		(*n* = 10)	
	*N*	%	*N*	%
*Gender*				
Male	8	80	8	80
Female	2	20	2	20
*Age*				
10.0–10.11	4	40	5	50
11.0–11.11	3	30	5	50
12.0–12.11	3	30	—	—

*Grade*				
4	2	20	4	40
5	3	30	4	40
6	5	50	2	20

**Table 5:** The comparison of the quadrant and subquadrant pretest scores between the control and experimental group on the academic activity (reading comprehension and written expression).

PRPP System: Thai Version		Mean rank		*Z*	Asymp.Sig (2-tailed)	Effect size (*r*)
Quadrant	Subquadrant	Control group	Experiment group			
		(*n* = 10)	(*n* = 10)			
*Perceive*		12.45	8.55	−1.49	.136	.33
	Attending	11.20	9.80	−.54	.59	.12
	Sensing	12.85	8.15	−1.91	.06	.43
	Discriminating	11.45	9.55	−.82	.41	.18

*Recall*		12.30	8.70	−1.38	.17	.31
	Recalling Facts	12.75	8.25	−1.84	.07	.41
	Recalling Schemes	11.05	9.95	−.43	.66	.10
	Recalling Procedures	9.10	11.90	−1.14	.25	.26

*Plan*		11.50	9.50	−.77	.44	.17
	Mapping	13.25	7.75	−2.19	.03^*∗*^	.49
	Programming	11.20	9.80	−.59	.56	.13
	Evaluating	11.20	9.80	−.58	.57	.13

*Perform*		11.05	9.95	−.43	.67	.10
	Initiating	11.75	9.25	−1.10	.27	.25
	Continuing	10.40	10.60	−.09	.93	.02
	Controlling	10.50	10.50	.00	1.00	.00

*Note*. ^*∗*^*p* < .05.

**Table 6:** The comparison of the quadrant and subquadrant pretest and posttest scores on the academic activity in the control group and experimental group.

PRPP System: Thai Version		Control group			Experimental group		
		(*n* = 10)			(*n* = 10)		
Quadrant	Subquadrant	*Z*	Asymp.Sig (2-tailed)	Effect size (*r*)	*Z*	Asymp.Sig (2-tailed)	Effect size (*r*)
*Perceive*		−1.69^b^	.09	.38	−2.56^a^	.01^*∗*^	.57
	Attending	−.62^b^	.54	.14	−1.85^a^	.07	.41
	Sensing	−2.69^b^	.01^*∗*^	.60	−2.72^a^	.007^*∗*^	.61
	Discriminating	−1.52^b^	.13	.34	−1.30^a^	.19	.29

*Recall*		−.42^a^	.67	.09	−2.39^a^	.02^*∗*^	.53
	Recalling Facts	−1.88^b^	.06	.42	−2.54^a^	.01^*∗*^	.57
	Recalling Schemes	−1.51^a^	.13	.34	−1.44^a^	.15	.32
	Recalling Procedures	−.85^a^	.40	.19	−2.46^b^	.01^*∗*^	.55

*Plan*		−1.18^b^	.24	.26	−2.50^a^	.01^*∗*^	.56
	Mapping	−.67^b^	.51	.15	−2.84^a^	.004^*∗*^	.64
	Programming	−.86^b^	.39	.19	−2.58^a^	.01^*∗*^	.58
	Evaluating	−1.67^b^	.10	.37	−2.51^a^	.01^*∗*^	.56

*Perform*		−.69^b^	.50	.15	−2.68^a^	.007^*∗*^	.60
	Initiating	−.27^b^	.79	.06	−1.41^a^	.16	.32
	Continuing	−1.00^b^	.32	.22	−2.72^a^	.007^*∗*^	.61
	Controlling	−.45^a^	.66	.10	−1.60^a^	.11	.36

*Note*. ^a^Based on negative ranks. ^b^Based on positive ranks. ^*∗*^*p* < .05.

**Table 7:** The comparison of quadrant and subquadrant posttest scores between the control and experimental group on the academic activity (reading comprehension and written expression).

PRPP System: Thai Version		Mean rank		*Z*	Asymp.Sig (2-tailed)	Effect size (*r*)
Quadrant	Subquadrant	Control group	Experiment group			
		(*n* = 10)	(*n* = 10)			
*Perceive*		15.50	5.50	−3.80	.000^*∗∗*^	.85
	Attending	13.40	7.60	−2.24	.03^*∗*^	.50
	Sensing	15.50	5.50	−3.93	.000^*∗∗*^	.88
	Discriminating	12.65	8.35	−1.85	.06	.41

*Recall*		10.25	10.75	−.19	.85	.04
	Recalling Facts	13.65	7.35	−2.46	.01^*∗*^	.55
	Recalling Schemes	9.55	11.5	−.75	.46	.17
	Recalling Procedures	7.70	13.30	−2.22	.03^*∗*^	.50

*Plan*		14.75	6.25	−3.24	.001^*∗*^	.72
	Mapping	14.50	6.50	−3.09	.002^*∗*^	.69
	Programming	14.50	6.50	−3.47	.000^*∗∗*^	.78
	Evaluating	14.80	6.20	−3.39	.000^*∗∗*^	.76

*Perform*		14.75	6.25	−3.26	.001^*∗*^	.73
	Initiating	10.50	10.50	.000	1.00	.00
	Continuing	15.40	5.60	−3.93	.000^*∗∗*^	.88
	Controlling	12.50	8.50	−1.62	.11	.36

*Note*. ^*∗∗*^*p* < .001. ^*∗*^*p* < .05.
